# Immune cell - derived microparticles contribute to the resistance of rheumatoid arthritis synovial fibroblasts to death receptor-mediated apoptosis

**DOI:** 10.1186/ar3616

**Published:** 2012-02-09

**Authors:** Mojca Frank, Meike Dahlhaus, Maria Filkova, Christoph Kolling, Beat A Michel, Diego Kyburz, Blaž Rozman, Renate E Gay, David Pisetsky, Steffen Gay, Astrid Jüngel

**Affiliations:** 1 Center of Experimental Rheumatology, University Hospital Zürich, Zürich, Switzerland; 2 Schultess Clinic, Zürich, Switzerland; 3 Department of Rheumatology, University Medical Centre Ljubljana, Ljubljana, Slovenia; 4 Medical Research Service, Durham Veterans Administration Medical Center, Durham, NC, USA

## Background

Immune cell-derived microparticles (MPs) are present at increased amounts in synovial fluid of rheumatoid arthritis (RA) patients [1] and can activate disease-relevant signalling pathways in RA synovial fibroblasts (SF) [2,3]. Increased resistance to apoptosis is one of the main characteristics of aggressive phenotype of RASF [4,5] and MPs have been shown to mediate both pro- and anti- apoptotic effects in different target cells [6,7]. The aim of the present study was to investigate the functional role of immune cell-derived MPs in modulating the apoptosis of SF in RA.

## Methods

MPs were isolated by the differential centrifugation from cell culture supernatants of U937 cells, untreated or stimulated with TNFα or poly(I:C) for 16 h. Flow cytometry was used to measure the counts and surface expression of CD4 and Fas on MP. Proinflammatory response of RASF induced by MPs was determined by measuring IL-6 protein levels by ELISA. Proliferation of OASF (n = 3) and RASF (n = 4) stimulated with MPs for 24 h was investigated by MTT Cell Proliferation Assay. Functional role of MPs (after 24 h treatment) in spontaneous apoptosis and apoptosis mediated by Fas Ligand (FasL) or TNFα-Related Apoptosis Inducing Ligand (TRAIL) was measured by flow cytometry using Annexin V/propidium iodide staining of RASF and OASF.

## Results

Poly(I:C)-induced MPs but not MPs from unstimulated U937 cells increased the production of IL-6 in RASF (mean ± SE: 1873 ± 325 pg/mL, p = 0.002, n = 9 and 476 ± 182 pg/mL, n = 6, respectively) when compared to unstimulated RASF (304 ± 61 pg/mL, n = 9). No changes in proliferation or spontaneous rate of apoptosis were observed in RASF or OASF stimulated with MPs. Treatment of RASF (n = 5) and OASF (n = 5) with FasL or treatment of RASF (n = 7) with TRAIL for 24 h significantly increased apoptosis of SF (p = 0.010; p = 0.036 and p = 0.016, respectively). Poly(I:C)-induced MPs inhibit FasL-induced apoptosis of RASF (% decrease ± SE: 40.2 ± 7.0%; p = 0.001; n = 5) and OASF (41.1 ± 9.5%; p = 0.036, n = 5) and decreased TRAIL-induced apoptosis of RASF (29.9 ± 6.8%, p = 0.093). In contrast, TNFα-induced MPs had no effect on Fas-induced apoptosis in SF (n = 3). MPs from untreated U937 cells did not influence FasL- or TRAIL-induced apoptosis of RASF (n = 5) and OASF (n = 4). Fas was not expressed on the surface of MPs, indicating that Poly(I:C)-induced MP did not act as a decoy to decrease the effective concentration of FasL in cell culture supernatants.

## Conclusions

Immune cells and SF can communicate via MPs. The impairment of the death receptor-induced apoptosis pathway mediated by immune cell-derived MPs may contribute to synovial hyperplasia and joint destruction in RA.
